# Deep Sequencing and Screening of Differentially Expressed MicroRNAs Related to Milk Fat Metabolism in Bovine Primary Mammary Epithelial Cells

**DOI:** 10.3390/ijms17020200

**Published:** 2016-02-17

**Authors:** Binglei Shen, Liying Zhang, Chuanjiang Lian, Chunyan Lu, Yonghong Zhang, Qiqi Pan, Runjun Yang, Zhihui Zhao

**Affiliations:** 1 College of Animal Science, Jilin University, 5333 Xi’an Road, Changchun 130062, China; binglei514@163.com (B.S.); lyzhang@jlu.edu.cn (L.Z.); cylu@jlu.edu.cn (C.L.); yyzhang@jlu.edu.cn (Y.Z.); 2 College of Animal Science and Veterinary Medicine, Heilongjiang Bayi Agricultural University, Daqing 163319, China; 18004598498@163.com; 3 National Key Laboratory of Veterinary Biotechnology and Laboratory Animal and Comparative Medicine Unit, Harbin Veterinary Research Institute, Chinese Academy of Agricultural Sciences, Harbin 150001, China; lianchuanjiang@caas.cn

**Keywords:** microRNA, Solexa sequencing, milk fat, primary mammary epithelial cell, dairy cow

## Abstract

Milk fat is a key factor affecting milk quality and is also a major trait targeted in dairy cow breeding. To determine how the synthesis and the metabolism of lipids in bovine milk is regulated at the miRNA level, primary mammary epithelial cells (pMEC) derived from two Chinese Holstein dairy cows that produced extreme differences in milk fat percentage were cultured by the method of tissue nubbles culture. Small RNA libraries were constructed from each of the two pMEC groups, and Solexa sequencing and bioinformatics analysis were then used to determine the abundance of miRNAs and their differential expression pattern between pMECs. Target genes and functional prediction of differentially expressed miRNAs by Gene Ontology and the Kyoto Encyclopedia of Genes and Genomes analysis illustrated their roles in milk fat metabolism. Results show that a total of 292 known miRNAs and 116 novel miRNAs were detected in both pMECs. Identification of known and novel miRNA candidates demonstrated the feasibility and sensitivity of sequencing at the cellular level. Additionally, 97 miRNAs were significantly differentially expressed between the pMECs. Finally, three miRNAs including bta-miR-33a, bta-miR-152 and bta-miR-224 whose predicted target genes were annotated to the pathway of lipid metabolism were screened and verified by real-time qPCR and Western-blotting experiments. This study is the first comparative profiling of the miRNA transcriptome in pMECs that produce different milk fat content.

## 1. Introduction

Milk fat traits of dairy cows are important for both milk processing and human health. The synthesis and secretion of milk fat is strictly regulated at the cellular level during the lactation cycle of dairy cows. Much research on molecular mechanisms of this process has been performed. The transcription factors *SREBP*s (Sterol regulatory element binding proteins), *PPAR* (Peroxisome proliferative activated receptor) and *LXR* (Liver X Receptor) are well known for their involvement in milk fat metabolism in mammary glands [1,2,3]. *DGAT1* (Diacylglycerol *O*-acyltransferase 1), *GHR* (Growth hormone receptor), *ABCG2* (ATP-binding cassette sub-family G member 2), *OPN* (Opsin receptor), *FASN* (Fatty acid synthase), and *SCD* (Acyl-CoA desaturase) were also identified as candidate genes or genetic markers that may impact milk fat traits through genome-wide association studies, functional genomics and comparative genomics analyses [4,5,6]. In addition to this classic transcriptional regulation pathway, members of noncoding RNAs, termed microRNA (miRNAs), have now been uncovered to be potent post-transcriptional regulators in fatty acid and cholesterol metabolism by targeting lipid metabolism genes. These miRNAs include: miR-33 [7], miR-122 [8], miR-370 [9], miR-378/378* [10], miR-27 [11], miR-143 [12], miR-335 [13], miR-103 [14] and so on. However milk fat synthesis is different from total lipid metabolism, it is predominantly synthesized and secreted in the mammary gland which is the most active tissue in the body with regard to milk fat metabolism. Milk fat metabolism in the mammary gland includes de novo synthesis of fatty acids, triglyceride synthesis, fat droplet formation, and fatty acid uptake and transport. While studies on the identification and characteristics of miRNA in mammary gland have mainly focused on different developmental stages or lactation cycles of ruminant animals [15,16,17], research on the comparative profiles of miRNAs in mammary glands that produce significantly different milk fat content have been scarce. Even fewer studies have been conducted using the mammary epithelial cells. However, Alsaweed *et al.* recently identified 293 and 233 miRNA species in the human breast milk cells and lipid fractions [18,19]. The miRNA content in maternal peripheral blood mononuclear cells was compared with that in plasma as well. The comparison results demonstrated that the mammary gland epithelium appeared to be the dominant origin of milk miRNA. That was consistent with the miRNA analysis in tammar wallaby milk [20]. These studies strongly suggest that miRNAs are primarily endogenously synthesized in the mammary epithelium, which can be the best model for screening small RNAs related to milk lipid metabolism.

Therefore, to determine how the synthesis or metabolism of lipid in milk is regulated at the miRNA level, small RNA libraries were constructed from each of the primary mammary epithelial cell (pMEC) cultures derived from Chinese Holstein dairy cows that produced extreme differences in milk fat percentage. An advantage of using pMECs for this study is that their differentiation potential is not diminished by an extended number of passages during *in vitro* culture, and they maintain the functions of lipid synthesis and secretion and are free of other cell types in the mammary gland [21,22] Solexa sequencing and bioinformatics analysis were then used to determine the abundance of miRNAs and their differential expression patterns between the pMECs. Differentially expressed miRNAs and their potential functions were subsequently predicted by GO and KEGG annotation. Finally, three miRNAs and their reverse-complementary target gene candidates that were annotated to the pathway of fatty acids metabolism were screened in mammary tissues from high and low milk fat percentage cows and identified by real-time q-PCR and Western-blotting experiments. To our knowledge, this study was the first comparative profiling of the miRNA transcriptome in pMECs that produce different milk fat percentages. These results can guide further studies of miRNA in mammary epithelial cells and their likely roles in milk fat metabolism in dairy cows.

## 2. Results

### 2.1. Cultures of Bovine pMECs and Comparisons of Triglyceride Content

The milk composition of 30 dairy cows is shown in Table S1. Mammary tissues from the most extreme (fat content difference) dairy cows #28 (pMEC-HH; 4.80 milk fat percentage and 3.39 milk protein percentage) and #1 (pMEC-LL; 2.75 milk fat percentage and 2.80 milk protein percentage) were used as the source of primary mammary epithelial cells (pMECs). The pMECs were obtained after two to three passages, and they had normal morphology, cytogenetics, growth and secretory characteristics, which have been described previously [23].

Triglycerides (TGs), which account for 95% of milk fat [24], can be used for the comparison of milk fat synthesis. We examined three passages and the results showed a significant difference between the two pMECs at passages two and three (Table 1). However, this difference gradually decreased as the number of passages increased, especially after the fifth passage. Accordingly, only primary mammary epithelial cells (cells in passage two) were used for Solexa sequencing.

**Table 1:** TG contents in two different bovine mammary epithelial cells (MEC, µmol/L).

	pMEC-HH	pMEC-LL
Passage 2	179.3441 ± 12.68155 ^A^	84.0345 ± 2.76037 ^A^
Passage 3	162.9136 ± 3.81292 ^A^	69.0142 ± 11.83466 ^A^
Passage 5	112.2417 ± 9.79188 ^B^	109.2433 ± 10.54467 ^B^

pMEC-HH: mammary epithelial cells cultured from high milk fat content tissue; pMEC-LL; mammary gland epithelial cells cultured from low milk fat content tissue. The triglycerides were analysed at different cell passages using a commercially available TG kit, and all results were expressed as means ± S.D. A different letter (A/B) in the same column means significantly different (*p* < 0.01).

### 2.2. Sequence Data Analysis

Detailed sequence data for the two libraries are displayed in Table S2. A total of 8.9 million and 11.2 million clean reads were obtained from the pMEC-HH and pMEC-LL small RNA libraries, respectively. Small RNA annotation is presented in Table S2A,B. The rRNAs and tRNAs were the majority of all non-coding RNA categories which accounted for the most components in protein synthesis. As well, 7,087,726 and 9,059,038 reads of candidate miRNAs were identified from pMEC-HH and pMEC-LL, respectively, and used for further analysis. The distribution of sequence lengths was normal and similar between the two libraries, and the predominant sequences were 22 nt in length (Table S2C,D). The number of common sequences between both libraries was much higher (98.62%) compared with that for unique small RNAs (11.98%), which suggested that the number of pMEC-HH-specific and pMEC-LL-specific sequences was much smaller compared with that of common sequences between the two libraries (Table S2E).

### 2.3. Identification of Known and Novel miRNAs in Bovine pMECs

**Figure 1:**
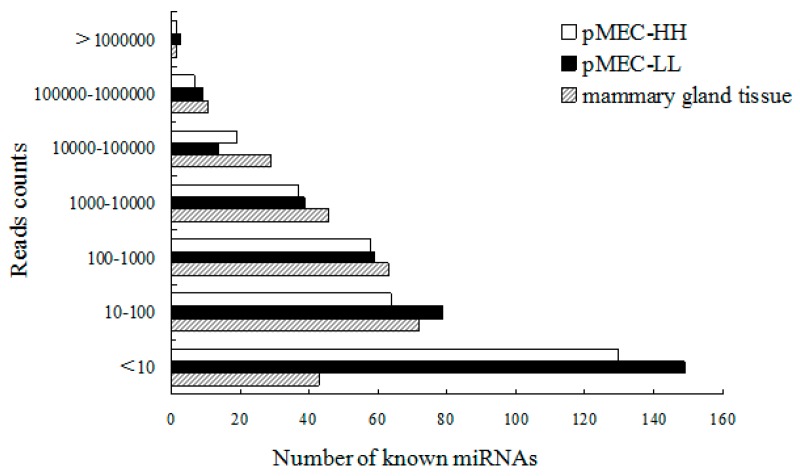
Sequencing reads distribution of known miRNAs in pMECs and mammary gland tissues. The distribution sequencing reads in the bovine lactating mammary gland was reported by Zhen *et al.* [15].

After successive filtering of these data sets, 292 known miRNAs were identified with 254 miRNAs appearing in both libraries. The total numbers of known miRNAs that identified in two libraries are shown in Table 2. However, 17 miRNA/miRNA* (miRNA* is from the opposite arm of the miRNA precursor) were detected only in pMEC-HH small RNA library and 21 miRNA/miRNA* were specifically expressed in pMEC-LL. More information about the conserved miRNAs in both libraries, including the miRNA sequence, miRNA precursor sequence and structures are listed in Table S3. The known miRNAs in our results were compared with the miRNAs identified in bovine lactating mammary gland tissues by Zhen *et al.* [15]. As expected, the total number was close (292 for pMECs and 283 for mammary gland) and the read counts distribution was also similar except for those of no more than 10 (Figure 1). The numbers in pMECs with read counts <10 (149) were much higher than those in lactating mammary gland (43), indicating that many miRNAs identified in pMECs were not detected in mammary tissue. Furthermore, 12 miRNAs with read counts exceeding 1000 in both pMEC libraries were also not found in mammary gland tissue. These included bta-miR-125b (16,807), bta-miR-16b (1023), bta-miR-92a (5157), bta-miR-378 (2098), bta-miR-940 (1435) and bta-miR-2284x (31,532).

**Table 2:** Identification of known bovine miRNAs in two different pMECs.

	miRNA	miRNA*	miRNA-5p	miRNA-3p	pre-miRNA
Known miRNAs in miRase 18.0	579	42	28	27	662
pMEC-HH	263	17	19	17	320
pMEC-LL	292	21	19	20	355

miRNA, mature sequences are predominant product originate from the predicted precursor; miRNA*, sequences are from the opposite arm of miRNA; miRNA-5p, sequences are from the 5’ arm; miRNA-3p, sequences are from the 3’ arm.

Having filtered the known non-coding RNAs, 116 novel bovine miRNAs were predicted. 32 novel miRNAs were expressed in both libraries. The secondary structures of these miRNAs are shown in Table S4. Unsurprisingly, the read numbers for all novel miRNAs were much lower than those for the majority of known miRNAs. For instance, the highest read numbers for the novel miRNAs miR-13 and miR-52 were only 1501 and 1995, respectively. But, the total reads of bta-miR-222 and bta-let-7a, which were conserved in many species, were nearly up to 1.9 million and 2.3 million, respectively.

### 2.4. Validation of miRNA Expression Patterns at Both Cellular and Tissular Levels

**Figure 2:**
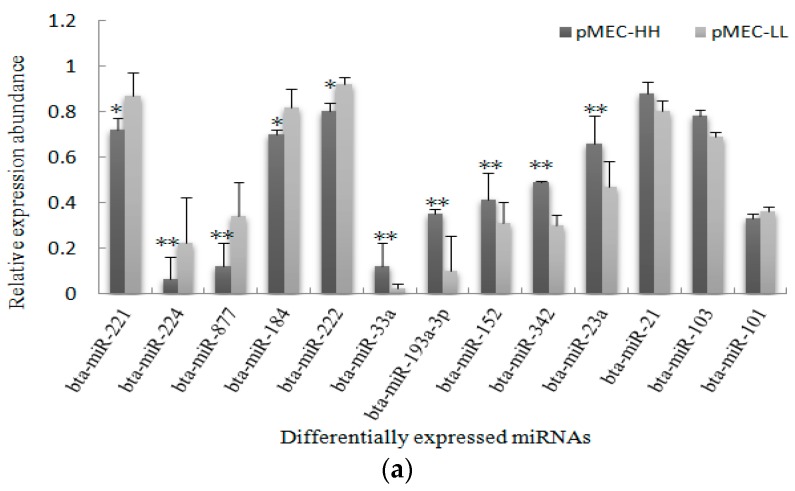

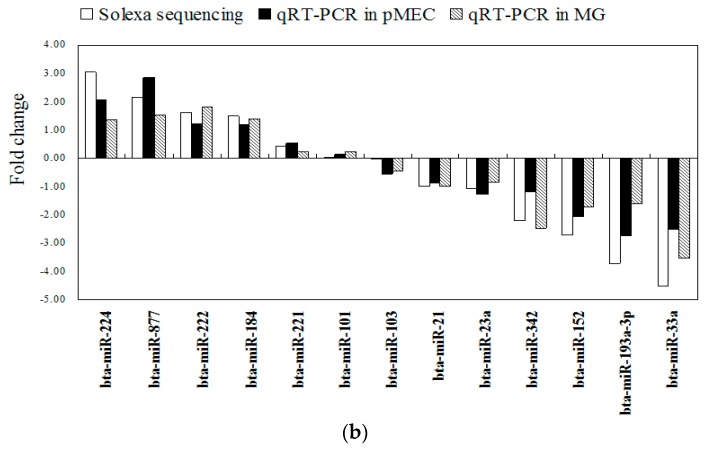
Validation of known miRNAs using stem-loop qPCR. (**a**) 13 known miRNAs were detected by stem-loop qPCR, using three replicates. The relative expression was calculated using the 2^−ΔΔ*C*t^ method after the threshold cycle (*C*_t_) and was normalized using the *C*_t_ of U6. The relative expression levels were presented as the 2^−ΔΔ*C*t^ means ± standard error. The error bars indicate the standard error of the 2^−ΔΔ*C*t^ mean values. *: 0.01 < *p* < 0.05, **: *p* < 0.01; (**b**) Comparison of expression patterns for known miRNAs (identified by Solexa sequencing) between pMECs and mammary gland (MG) tissues. *X*-axis: the miRNA types; *Y*-axis: expression differences of miRNAs in high and low fat samples. Because the methods used to calculate differential expression by stem-loop qPCR differs from that used by Solexa sequencing, significance analysis was not performed, and the figure only shows the general trend of miRNA expression.

The present study showed that the expression levels of 13 randomly selected miRNAs were identical to that found by Solexa sequencing (Figure 2a), and furthermore, a high degree of consistency was observed between the results of qPCR from pMECs and mammary gland tissues (Figure 2b). The stem-loop qPCR results confirmed similar miRNA expression patterns between cells and fresh tissues.

### 2.5. Differential Expression of miRNAs and Functional Analysis of Their Predicted Target Genes

The differential expression profiles of known miRNAs between the two libraries are shown in Table S5. Ninety-seven miRNAs were differentially expressed at significance level (*p* < 0.05). Among these, 39 were more highly expressed in pMEC-LL; and 58 were expressed at a lower level in pMEC-LL, suggesting that these miRNAs may have regulatory roles in the synthesis, secretion and regulation of milk fat in mammary gland tissue. The top ten different fold change miRNAs are listed in Table 3.

**Table 3:** 10 greatest fold change miRNAs between the two pMECs.

miR-Name	pMEC-HH-std	pMEC-LL-std	Fold-Change	*p* Value	FDR	Sig-Label
bta-miR-124a	0.0100	14.8710	10.5383	5.50 × 10^−^^43^	0.0813	**
bta-miR-224	1.9114	15.8505	3.05183	1.22 × 10^−^^27^	0.2259	**
bta-miR-452	11.4682	70.0808	2.6114	6.56 × 10^−^^100^	0.3946	**
bta-miR-877	39.8015	175.7807	2.1429	8.77 × 10^−^^197^	0.2389	**
bta-miR-2344	40.4761	169.7254	2.0681	8.17 × 10^−^^182^	0.8923	**
bta-miR-29b	588.2531	97.5966	−2.5915	0	0.9213	**
bta-miR-152	1231.1490	189.5831	−2.6991	0	0.4573	**
bta-miR-30b-5p	20.9127	1.8700	−3.4833	1.68 × 10^−^^43^	0.1364	**
bta-miR-193a-3p	265.4560	20.2139	−3.7151	0	0.8523	**
bta-miR-33a	32.3809	1.4248	−4.5063	1.09 × 10^−^^80^	0.4039	**

pMEC-HH-std: The normalized expression level of miRNA in pMEC-HH library; pMEC-LL-std: The normalized expression level of miRNA in pMEC-LL library; fold-change: log2 pMEC-LL-std/pMEC-HH-std; FDR: False Discovery Rate; **: fold change (log2) > 1 or fold change (log2) < −1, and *p* value < 0.01.

**Figure 3:**
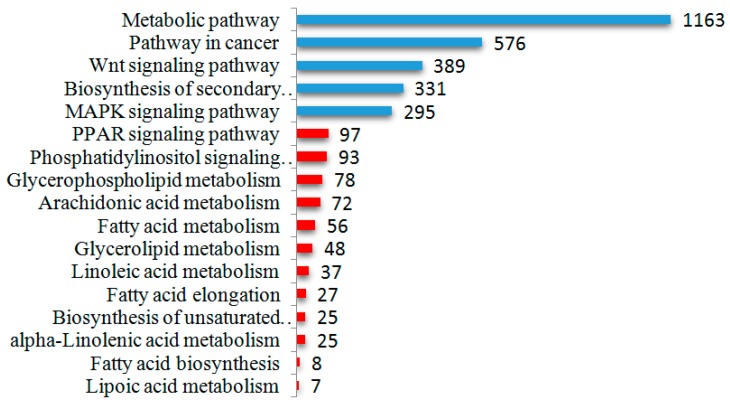
The number of miRNA predicted target genes mapped by pathway. Blue column: the significantly enriched pathway of target genes. Pink column: the annotated pathway related to the lipid metabolism.

A total of 88,334 mRNA transcripts were annotated and predicted as candidate genes for 97 differentially expressed known miRNAs (Table S6). The most enriched GO terms of three ontologies are listed in Figure S1. The cellular component GO analysis showed that these target genes were related to the cell part, intracellular part and membrane-bounded organelle, functioning in protein binding, catalytic activities and zinc ion binding. Cellular process, single-organism process and single-organism cellular process were the most significantly enriched in terms of biological process. Intriguingly, metabolic process, organic substance metabolic process, primary metabolic process and regulation of metabolic process are also significantly enriched GO terms, suggesting that the dominant target genes of differentially expressed miRNAs are involved in the metabolism process. KEGG pathway annotation showed that 9887 target genes were annotated to 305 biological processes (Table S7), and most of the target genes were involved in metabolism (11.76%) or the biosynthesis of secondary metabolites (3.35%, Figure 3).The second most common pathway was the pathway in cancer (5.83%), which may reflect the fact that mammary epithelial cells during the lactation period maintain a highly proliferative state. In addition, over-represented miRNA targets were involved in the Wnt signal transduction pathways, which are known to be associated with adipogenesis and developmental processes [25].

### 2.6. Screening and Identification of miRNAs Related to Milk Fat Metabolism in Dairy Cattle

**Figure 4:**
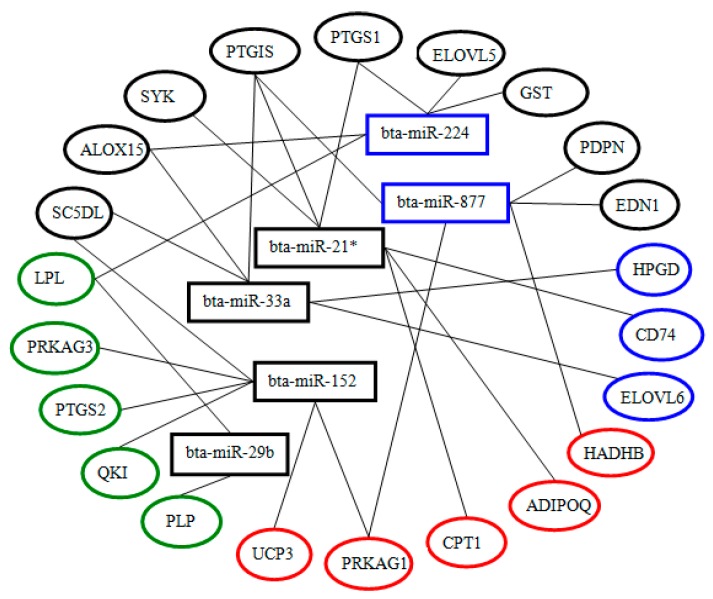
Six miRNAs and their target genes that are involved in milk fat metabolism in bovine mammary gland. The oval color represents the pathway that the target genes involved. Blue oval: Unsaturated fatty acid synthesis. Green oval: fatty acid biosynthesis. Red oval: fatty acid metabolism. Black oval: the targets involved in more than one metabolic pathway. The colored boxes represent the expression trend. Black rectangle: down-regulated miRNAs in pMEC-LL. Blue rectangle: up-regulated miRNAs in pMEC-LL.

Eight miRNAs were first selected randomly from 97 differentially expressed known miRNAs. By analyzing their potential target genes that annotated to four pathways related to lipid metabolism, six miRNAs were filtered including bta-miR-33a, bta-miR-21*, bta-miR-29b, bta-miR-152, bta-miR-224 and bta-miR-887. The first four were down-regulated in both pMEC-LL and mammary gland tissues with low milk fat percentage and in contrast, the last two were up-regulated. The predicated 22 target genes for these six miRNAs are shown in Figure 4 and Table S8.

**Figure 5:**
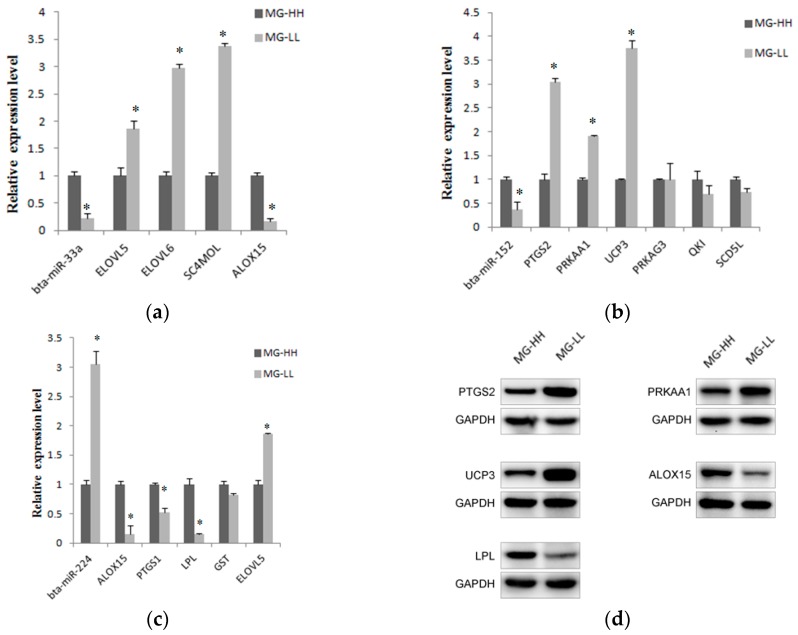
Relative expression analysis of miRNAs and their predicted target genes. (**a**) Bta-miR-33a was down-regulated, which was opposite to the expression level of *ELOVL5*, *ELOVL6* and *SC4MOL* in MG-LL; (**b**) Bta-miR-152 was down-regulated in MG-LL and the significantly up-regulated genes were *PTGS2*, *PRKAA1* and *UCP3*; (**c**) Bta-miR-224 was up-regulated in MG-LL and the corresponding down-regulated genes were *ALOX15*, *PTGS1*, *LPL* and *GST*; (**d**) Western-blotting results of five target genes. All of the results are expressed as the mean ± S.D. of three replicates. * *p* < 0.05; GAPDH is internal control.

To gain further insight into the reverse and complementary relationship between the six miRNAs and 22 possible target genes, real-time q-PCR and Western-blotting experiments were carried out. The expression levels are shown in Figure 5. From the results, most genes were found to be differentially expressed between MG-LL (mammary gland with low milk fat content) and MG-HH (mammary gland with high milk fat content). According to the causality of miRNA expression patterns and their target gene, three miRNAs showed a significant inverse correlation in expression with their corresponding targets. They are bta-miR-33a, bta-miR-152 and bta-miR-224.

## 3. Discussion

The premise of the experiment was to obtain pMECs from cows with extreme differences in milk fat production. The largest component of milk fat is triglycerides [26]. The fatty acid content and composition of milk triglycerides is markedly affected by breeds, stage of lactation, dietary manipulations and individuals. Therefore, in the current study, mammary gland tissues used for pMEC cell cultures were harvested from cows of the same breed, lactation stage and feeding method but with significant differences in milk fat and milk protein contents. The pMECs we obtained showed the characteristic “cobblestone” morphology of epithelial cells. This was further confirmed by the protein expression of cytokeratin 18 which is a specific marker for epithelium. Most isolated epithelial cells were positive-stained and the fibroblast culture and negative control showed no cytokeratin staining. This indicates the specific epithelium character of cell cultures that we isolated [23]. However, although the predominant cell type in tissue cultures was of epithelial origin, it is likely that the cell cultures still contained some adipose cells, which were reduced after multiple steps of purification. Furthermore, to confirm whether the cell cultures maintained the extreme difference in lipid synthesis, TG contents were continuously detected in different cell passages. The results showed that the difference between pMEC-HH and pMEC-LL remain intact during the first 2–5 passages. D. Sorg *et al.* studied the cholesterol efflux ability of 2nd, 3rd, 5th and 7th passage cultures; the results showed no significant variation and confirmed the sustained functionality of pMECs in cultures [27]. However, the difference of TG content gradually diminished as the number of cell passages increased in the present study, especially after the fifth passage. Accordingly, only primary mammary epithelial cells (cells in passage two to five) were used. One possible reason is the *in vivo* conditions can never be mimicked perfectly and cells sense the chemical and physical surroundings via adhesion receptors. So it is understandable that some biological character changes may occur after continuous cell passages. Generally speaking, pMECs *in vitro* are similar in genetics, structure, and functions to the initial passages, and therefore constitute the best model system for *in vitro* comparative studies.

Besides, by comparing the profile pattern of conserved miRNAs in pMEC with those in mammary gland tissue, the results indicate that screening for miRNAs associated with milk fat synthesis at the cellular level was feasible. Alsaweed et al identified 754 miRNAs in the cellular and lipid fraction of human milk by the Taqman OpenArray Panel system [18,19,28]. The results demonstrated milk lipid and cellular miRNAs are primarily synthesized in the mammary gland epithelial cells. The bta-miR-224, bta-miR-152 and bta-miR-33a, which were differentially expressed between pMEC-HH and pMEC-LL were also identified in abundance in the human lipid fraction [29], human skim milk [30], human colostrum [30], as well as in bovine skim milk and colostrum [31], suggesting that they play important roles in the lipid metabolism and/or synthesis in the mammary gland. On the other hand, miR-370 [9], miR-758 [32] and miR-106b [33], which were reported to play roles in the regulation of lipoprotein metabolism, were not detected in bovine pMECs. This may be because the synthesis of fatty acids is markedly different between mammalian species. The precursors used and the mechanism of fatty acid synthesis in ruminants are greatly different from those of non-ruminant animals [34]. Secondly, the expression and regulation of miRNAs have the characteristics of being tissue- and development-specific. Wang *et al.* [35] found that there are major differences in the expression patterns of miRNAs during different stages of lactation. In our study, the cows used for cell isolation were all in mid-lactation. Therefore, whether the low-level novel miRNAs or undetected known miRNAs are expressed in pMECs derived from mammary glands at different lactation remains to be further investigated. Moving forward, we suggest that more basic research into lipid metabolism in different cell lines, especially cell cultures derived from mammary glands at different developmental stages, is essential.

The milk fat metabolism pathways in mammary gland are distinct from the other tissues of dairy cows. We detected 97 significantly differentiated expression miRNAs. miR-33a, a member of the human miR-33 family, is the most well-known miRNA involved in regulating cholesterol synthesis and fatty acids β-oxidation. Inhibition of miR-33a and miR-33b *in vitro* up-regulates fatty acid oxidation and the hepatocyte insulin response [7]. In the present study bta-miR-33a was expressed at a significantly lower level in pMEC-LL. This suggests that miR-33a might also play a regulatory role in milk fat metabolism in the bovine mammary gland. Another highlighted miRNA was bta-miR-29b, which was down-regulated in pMEC-LL as well. Ting *et al.* [36] concluded that miRNA-29a could target to bind LPL (lipoprotein lipase) specifically in the oxLDL-stimulated dendritic cells and further affect the secretion of pro-inflammatory cytokines and expression of scavenger receptors. This revealed the targeting relationship between miRNA-29 and LPL. However, whether they are regulators involved in milk fat metabolism still needs further clarification.

By performing pathway analysis, 22 predicted genes of six preliminarily filtered miRNAs were identified as involved in the processes of unsaturated fatty acid biosynthesis, fatty acid biosynthesis and fatty acid metabolism. It should be noted that one target gene can correspond to more than one miRNAs. For example, PTGIS (Prostaglandin I2 synthase) was located at bta-miR-33a, bta-miR-21* and bta-miR-224 at the same time. LPL was predicted as a target gene both for bta-miR-29b and bta-miR-877. These examples imply that not only can one miRNA can target several genes, but that one gene may be regulated by two or more miRNAs as well. That is to say that miRNAs are integrated into complex genetic networks that regulate milk fat metabolism. Also, there were nine target genes (genes in black ovals, Figure 4) that participate in more than one process related to milk fatty acid metabolism. PTGIS, for instance, is a critical enzyme involved in both fatty acid metabolism and unsaturated fatty acid biosynthesis.

By comparing the expression level of miRNAs and predicted target genes both at mRNA and protein levels, we finally screened out 3 miRNAs that have the potential to regulate milk lipid metabolism. The first one was bta-miR-33a whose predicted targets were ELOVL5, ELOVL6 and SC4MOL. The ELOVL (elongation of very-long-chain fatty acids, ELOVL) family has seven members which mainly participate in the mammalian long chain fatty acids synthesis and are all rate-limiting enzymes [37]. ELOVL5 and ELOVL6 usually catalyzed the elongation and biosynthesis of monounsaturated fatty acids(C:16-C:18) in cooperation. Hoekstra (2012) studied the low-density lipoprotein (LDL) receptor gene in knockout mice and concluded that the expression of miR-302 inhibited the expression of ELOVL6 [38]. As expected, ELOVL5 and ELOVL6 showed the same tendency of high expression in MG-LL in the present research; this indicated that both genes have the possibility of affecting the lipid synthesis by the regulation of bta-miR-33a. SC4MOL (sterol-C4-methyl oxidase-like, SC4MOL) is also an important gene involved in fatty acids synthesis. He *et al.* (2012) revealed that knocking out SC4MOL can increase cholesterol content [39]. The present q-PCR results detected that SC4MOL gene was up-regulated in the MG-LL and may function in the synthesis of fat and cholesterol which was also in accordance with the conclusion of He. In short, although the regulatory function of bta-miR-33a has been acknowledged in lipid metabolism, there are great differences in diverse tissues, organics and physiology conditions between general lipid and milk fat in mammary gland. Therefore, except for *SREBP1*, *ABCA1*, *AMPK*, *HADHB*, *ABCG1*, the target gene candidates *ELOVL5*, *ELOVL6* and *SC4MOL* which were up-regulated in MG-LL had the same reverse-complementary relationship with bta-miR-33a.

In terms of predicted targets of bta-miR-152, we found that they were all key genes in the pathway of fatty acids metabolism. PRKAA1 (protein kinase, AMP-activated, α 1 catalytic subunit, PPKAA1) for instance, is a cellular energy sensor that maintains energy homeostasis within the cell and is activated when the AMP/ATP ratio increases [40]. PRKAA1 can be activated during lactation which is a significant energy and substances consuming process, and then promote the oxygenolysis of general body fat to compensate for the tremendous consumption of energy during lactation, meanwhile inhibiting energy consuming processes, such as Glyconeogenesis and general fat synthesis [41,42]. The current study demonstrated the high expression of PRKAA1 in MG-LL, which means the inhibition of Glyconeogenesis; but most substance resources and 85 percent of energy were all dependent on the process of Glyconeogenesis during the lactation of dairy cows. Therefore we might conclude that the down-regulation of bta-miR-152 caused the up-regulation of PRKAA1, inhibited the Glyconeogenesis and finally decreased the milk fat biosynthesis in mammary gland. Another up-regulated target gene was PTGS2 (prostaglandin-endoperoxide synthase 2, PTGS2), encoding the induced enzyme cyclooxygenase-2 (COX-2) and mediating prostaglandin synthesis which is always highly expressed under inflammation, wound healing or tumorigenesis [43]. Recent studies about PTGS2 on livestock largely focused on the correlation of Single Nucleotide Polymorphisms and reproductive traits [44]. Also, UCP3 (uncoupling proteins, UCPs) is an uncoupling protein and marker for brown adipose tissue differentiation. The percentage of fat in adipose tissue with over expression of UCP was obviously lower than that of the control group, that is the UCP gene can decrease the content of milk fat at the tissue level [45].

As for bta-miR-224, its target gene candidates were GST, ALOX15 and PTGS1. GST (Glutathione *S*-transferase, GST) is an important bioactive peptide with the function of detoxification, antioxidation and participation in metabolic regulation. Moreover, there is research that indicates that the GST gene is involved in the elongation and synthesis of C:20 monounsaturated fatty acids [46]. Therefore, the up-regulated bta-miR-224 in MG-LL might inhibit the synthesis of fatty acids by combining with GST whose expression level was decreased in MG-LL. Another gene, LPL, has been studied for over hundreds of years. Recent research also has on several occasions identified a targeting relationship between miR-29 and LPL [47,48]. However, based on the present results, it is impossible for the down-regulated LPL to regulate lipid synthesis by combining with bta-miR-29b which was also down regulated in MG-LL. As a result, we hypothesized that instead of bta-miR-29, bta-miR-224 can be a new miRNA that affect milk fat metabolism by combining to the predicted target gene LPL. ALOX15 (arachidonate5-lipoxygenase-activating protein, ALOX15) and PTGS1 (prostaglandin-endoperoxide synthase 1, PTGS1) were also down-regulated significantly in MG-LL compared with that in MG-HH; through analyzing their function it was found that ALOX15 mainly regulates the lipid metabolism by involving to the synthesis of polyunsaturated fatty acids; PTGS1 is a gene coding cyclooxygenase1 (COX-1) which is different from PTGS2 and widely expressed in different tissues to produce endothelial prostacyclin. Their functions and regulating mechanism were mainly observed in diverse human diseases, such as coronary artery conditions, cardiomyopathy, chronic myeloid leukemia, obesity and diabetes or tumor-suppressive [49,50,51]. The function in milk lipid metabolism is still unclear and needs further research.

Taken together, three miRNAs were screened which may be regulators in milk fat metabolism by affecting their predicted target genes; they were bta-miR-33a, predicted target gene *ELOVL5*, *ELOVL6* and *SC4MOL*; bta-miR-152, predicted target gene *PTGS2*, *PRKAA1* and *CUP3*; bta-miR-224, predicted target gene *LPL*, *GST*, *ALOX15* and *PTGS1*.

## 4. Materials and Methods

### 4.1. Ethics Statement

All studies involving dairy cows were conducted according to regulations approved by Jilin University animal care and use committee (Permit number: SYXK (Ji) 2008-0010/0011). Animals were allowed access to feed and water ad libitum and were humanely sacrificed as necessary.

### 4.2. Animals and Sample Collection

Thirty healthy Chinese Holstein dairy cows, which were at the same stage of mid-lactation (181 ± 46 day), lactation amount (21.67 ± 3.60 kg/day), and the same parity (1.75 ± 1.07) were used in the present study. Diet composition was designed with reference to the National Research Council recommended standard (NRC, 2001) [52] and the forage: concentrate ratio was 40:60. Milk fat ratio and protein content were monitored twice daily for 10 days using a milk composition analyzer (Lactoscan SP, Milkatronics Ltd., Nova Zagora, Bulgaria). Six cows with the highest (mean ± S.D.) and lowest (mean ± S.D.) milk fat percentage (*n* = 3 for each group) were euthanized after 10 days on a common diet. The mammary tissues were randomly harvested and placed in an incubation box at 37 °C in physiological saline with 5% (*v*/*v*) penicillin and streptomycin for cell culture, or immediately frozen in liquid nitrogen and kept at −80 °C for qPCR analysis.

### 4.3. Cell Culture, Triglyceride Content Detecting and RNA Extraction

Tissue samples were removed from the incubation box and separated from adipose and connective tissues surgically. Then samples were minced using surgical scissors and transferred to a centrifuge tube containing PBS solution. After repetitive pipetting, the tissue samples were allowed to settle for 3–5 min. The supernatant was discarded and the tissue-washing step was repeated 3–5 more times before tissues were transferred to cell culture dishes (Falcon, Franklin Lakes, NJ, USA) with basal medium. The basal medium containing DMEM/F12 (GIBCO, Invitrogen, Carlsbad, CA, USA) supplemented with 10% (*v*/*v*) fetal bovine serum (GIBCO) and 1% (*v*/*v*) penicillin and streptomycin (Tiangen, Beijing, China) and 1% (*v*/*v*) epithelial growth factor. The cell culture dishes were then incubated at 37 °C at 5% CO_2_ incubator (Thermo, Marietta, OH, USA). The culture media was replaced with fresh media every 48 h until the culture dishes were full of cells. Cells were detached with 0.25% tripson, 0.02% EDTA (GIBCO) and transferred to new culture dishes, which were used to remove fibroblasts. Subsequently, the pure epithelial cells were obtained by continuous culture and digestion for 3–5 times.

Methods for the basic identification of bovine pMEC have been described previously [23]. TG content in the pMECs from the mammary glands with high milk fat ratio (named pMEC-HH) and low milk fat ratio (named pMEC-LL) was measured using a Triglyceride Determination Kit (Sigma-Aldrich, St. Louis, MO, USA) according to the manufacturer’s protocol. Total RNA from the two different pMECs was isolated using Trizol reagent (Invitrogen) and was prepared for Solexa sequencing according to the Illumina TruSeq small RNA Sample Preparation protocol.

### 4.4. Solexa Sequencing and Bioinformatic Analysis of Small RNAs

Small RNA (18–30 nt) libraries were constructed by the Beijing Genomics Institute (BGI) (Shenzhen, China) using an Illumina Genome Analyzer according to the manufacturer’s instructions. The filtered sequences were matched to the miRNAs in miRBase 18.0 (http://www.mirbase.org/) to identify know miRNAs and annotated stem-loop sequences. To predicted novel miRNAs, the flanking genome sequence of unique small RNAs was folded using the Mireap program developed by BGI (http://sourceforge.net/projects/mireap). The parameters for novel miRNA prediction are listed as follows: minimal miRNA sequence length is 18 nt; maximal miRNA sequence length is 26 nt; minimal miRNA reference sequence length is 20 nt; maximal miRNA reference sequence length is 24 nt; minimal depth of Drosha/Dicer cutting site is 3 nt; maximal copy number of miRNAs on reference is 20 nt; maximal free energy allowed for a miRNA precursor is 18 kcal/mol; maximal space between miRNA and the opposite arm of the miRNA (miRNA*) is 35 nt; minimal base pairs of miRNA and miRNA* are 14 nt; maximal bulge of miRNA and miRNA* is 4 nt. Finally, the conserved miRNAs between the two libraries were compared for the identification of differentially expressed known miRNAs.

### 4.5. miRNA Validation via Stem-Loop Real-Time qPCR Both at the Cellular and Tissular Level

To validate the Solexa sequencing results, 13 known miRNAs were selected randomly and analyzed by stem-loop real-time qPCR. To validate the consistency of miRNA profile patterns between pMECs and tissues, total RNA from the mammary gland tissues of 6 dairy cows that produce the highest and lowest milk fat percentage were isolated using a mirVana miRNA Isolation Kit (Ambion, Austin, TX, USA). These RNAs, as well as RNA from the most extreme pMEC-HH and pMEC-LL cells, were then analyzed by stem-loop RT-PCR followed by SYBR Green Real-time PCR. The selected mature miRNAs and primer sequences are shown in Table S9.

### 4.6. Target Gene and Function Prediction of Differentially Expressed MicroRNAs

Targets of the differentially expressed miRNAs were predicted by miRanda v3.3a and Target Scan 7.0 using 3′ UTR sequences retrieved from the University of California Santa Cruz (UCSC) Table Browser (http://genome.ucsc.edu/cgi-bin/hgTables). The rules used for target prediction are based on those suggested by Allen *et al.* [53] and Schwab *et al.* [54]. The GO (Gene Ontology) and the KEGG (Kyoto Encyclopedia of Genes and Genomes) analyses were performed to reveal the functions of these target gene candidates. Pathways related to fatty acid metabolism were emphasized to screen for miRNA with potential function in milk fat regulation.

### 4.7. Screening of miRNAs Related to Milk Fat Metabolism in Dairy Cattle

Eight miRNAs were selected randomly from the differentially expressed miRNAs between pMEC-HH and pMEC-LL. They included bta-miR-193a-3p, bta-miR-33a, bta-miR-21*, bta-miR-152, bta-miR-29, bta-miR-224, bta-miR-222 and bta-miR-877. By scanning the KEGG results these miRNAs were filtered according to their predicted target genes identified and predicted involvement in lipid metabolism. The focused pathways included fatty acid biosynthesis, fatty acid elongation, glycerolipid metabolism and biosynthesis of unsaturated fatty acid.

### 4.8. qPCR of Predicted Genes Involved in Lipid Metabolism

The expression levels of twenty-two target genes were conducted by qRT-PCR analysis. The primer sequences of predicted target genes were listed in Table S10. Real-time qRT-PCR was performed using the One Step PrimeScript^®^ cDNA Synthesis Kit (TAKARA, Beijing, China) and using 12.5 µL of SYBR^®^Premix Ex TaqTM II (2×) (TAKARA), 1 µL of PCR sense primer (10 µM), 1 µL of PCR antisense primer (10 µM), and 2 μL of five-fold cDNA template dilution in a 25-µL system with the following procedures: 95 °C for 30 s followed by 40 cycles at 95 °C for 15 s and finally 60 °C for 30 s. Glyceraldehyde-3-phosphate dehydrogenase (GAPDH) was used as the internal house-keeping control gene.

### 4.9. Western Blot Analysis of Predicted Proteins Involved in Lipid Metabolism

Five target proteins were selected for Western blot analysis. Proteins were extracted from six frozen tissue samples. Briefly, tissues total proteins were isolated using a mammalian protein isolation kit (Qiagen, Beijing, China). Then 50 μg of total lysate was separated in 10% polyacrylamide gels and transferred onto polyvinylidene difluoride (PVDF) membranes. After blocking in 5% non-fat dry milk in Tris-buffered saline and Tween 20 (TBS-T), the membranes were incubated with the following antibodies (Santa Cruz, Santa Cruz, CA, USA): LPL (sc32382), PRKAA1 (sc19126), UCPS (sc31387), ALOX5AP (sc28815), PTGS1 (sc7950) and GAPDH (sc25778,) overnight at 4 °C. Samples were then incubated with horseradish peroxidase (HRP)-conjugated anti-rabbit antibody (sc2347; Santa Cruz) for 1 h at 37 °C. Signal detection was done using an ECL Western Blotting kit (Amersham Pharmacia Biotech, Piscataway, NJ, USA).

### 4.10. Statistical Analysis

Statistical analysis was done using SPSS 13.0 statistical software. The data are shown as the mean ± S.D. A Student’s *t*-test was used for comparison of two independent groups. A one-way ANOVA analysis of variance was used to compare multiple groups. *p* < 0.05 was considered to indicate statistical significance.

## 5. Conclusions

We report the construction of two miRNA libraries at the cellular level that featured a difference in the capability of milk fat synthesis. Using the Solexa sequencing method. 97 significantly differentially expressed miRNAs between the two small RNA libraries were detected. Also, a consistency of miRNAs expression patterns between the primary mammary epithelial cells and mammary gland tissues was observed. More importantly, three miRNAs including bta-miR-33a, bta-miR-152 and bta-miR-224 were further validated that have potential regulatory functions in milk fat metabolism together with their predicted target genes. The three miRNAs can be used for further functional identification. To our knowledge, this is the first comparative expression pattern study in different mammary gland epithelial cells aimed at the screening of bovine miRNAs associated with milk fat metabolism.

## Supplementary Materials

Supplementary materials can be found at http://www.mdpi.com/1422-0067/17/2/200/s1.
