# Supplementary material for: Deep Sequencing and Screening of Differentially Expressed MicroRNAs Related to Milk Fat Metabolism in Bovine Primary Mammary Epithelial Cells
Source: Int J Mol Sci. 2016 Feb 17;17(2):200. doi: 10.3390/ijms17020200 (PMC4783934; doi:10.3390/ijms17020200)
Supplement: Supplementary file 1 [file ijms-17-00200-s001.zip › ijms-109791-Supplementary Materials/ijms-109791-Supplementary Materials.pdf]

Supplementary Materials: Deep Sequencing and Screening of Differentially Expressed microRNAs Related to Milk Fat Metabolism in Bovine Primary Mammary Epithelial Cells

Binglei Shen, Liying Zhang, Chuanjiang Lian, Chunyan Lu, Yonghong Zhang, Qiqi Pan, Runjun Yang and Zhihui Zhao

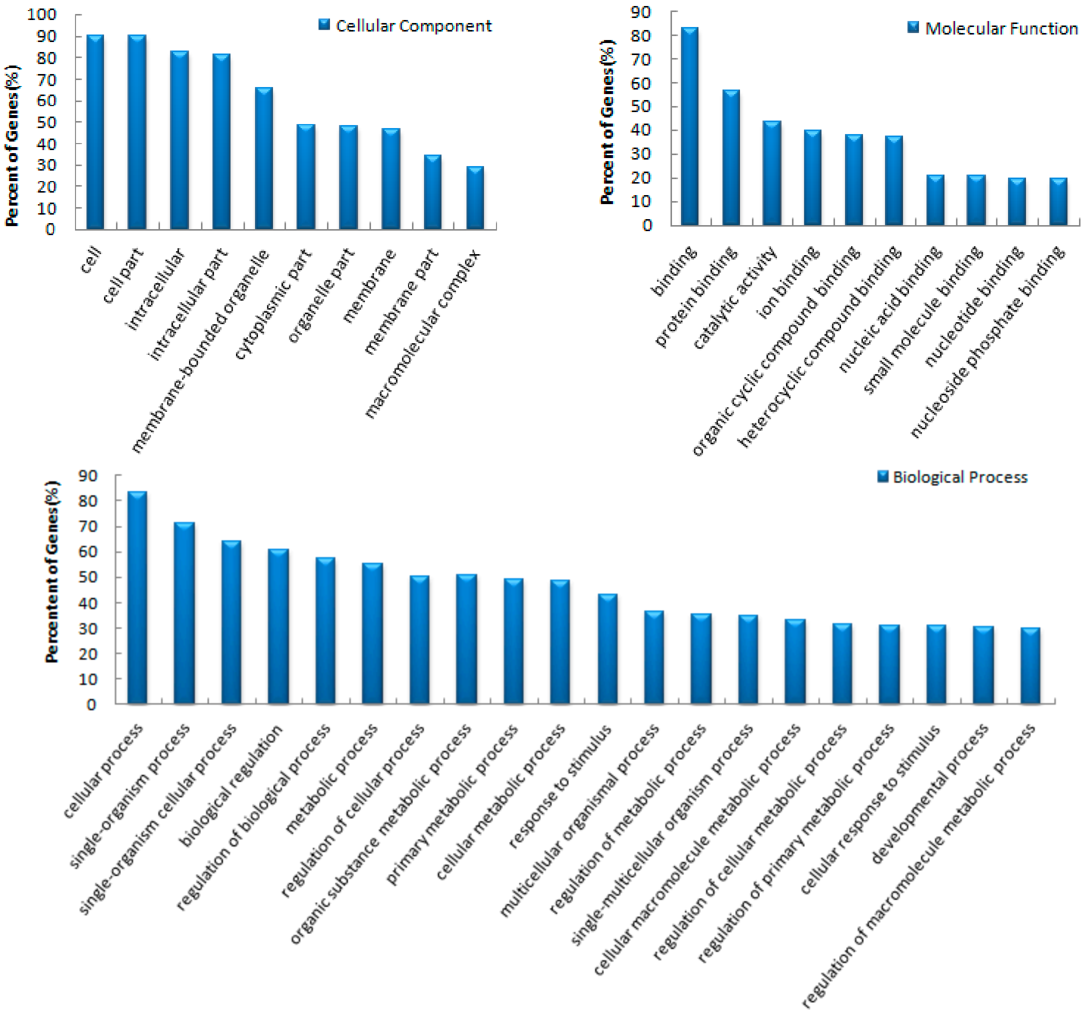

Figure S1. GO analyses of predicted targets of differentially expressed miRNAs. The top twenty categories enriched in biological processes and top ten categories enriched in both the cellular components and molecular functions.

Table S8. Six screened miRNAs whose target gene candidates were annotated to the pathway related to fatty acid metabolism.

Gene Symbol	Gene Description
	bta-miR-33a
	Unsaturated fatty acid biosynthetic process
ELOVL5	ELOVL family member 5
ALOX15	arachidonate 15-lipoxygenase
ELOVL 6	hypothetical LOC533333
SC4MOL	sterol-C4-methyl oxidase-like
	bta-miR-21*
	Unsaturated fatty acid biosynthetic process
CD74	CD74 molecule
PTGIS	prostaglandin I2 (prostacyclin) synthase
PTGS1	prostaglandin-endoperoxide synthase 1
SYK	spleen tyrosine kinase
	Fatty acid biosynthetic process
PTGIS	prostaglandin I2 (prostacyclin) synthase
PTGS1	prostaglandin-endoperoxide synthase 1
SYK	spleen tyrosine kinase
	Fatty acid metabolic process
ADIPOQ	adiponectin, C1Q and collagen domain containing
CPT1	carnitine palmitoyltransferase 1B (muscle)
	bta-miR-152
	Fatty acid biosynthetic process
PTGS2	prostaglandin-endoperoxide synthase 2
PRKAG3	protein kinase, AMP-activated, gamma 3 non-catalytic subunit
QKI	quaking homolog, KH domain RNA binding
SC5DL	sterol-C5-desaturase-like
	Fatty acid metabolic process
PRKAA1	protein kinase, AMP-activated, alpha 1 catalytic subunit
UCP3	uncoupling protein 3 (mitochondrial, proton carrier)
	bta-miR-224
	Unsaturated fatty acid biosynthetic process
ALOX15	arachidonate 5-lipoxygenase-activating protein
GST	microsomal glutathione S-transferase 2
PTGS1	prostaglandin-endoperoxide synthase 1
	Fatty acid biosynthetic process
ELOVL 5	ELOVL family member 5, elongation of long chain fatty acids
ALOX15	arachidonate5-lipoxygenase-activating protein
LPL	lipoprotein lipase
GST	microsomal glutathione S-transferase 2
PTGS1	prostaglandin-endoperoxide synthase 1
	bta-miR-877
	Unsatured fatty acid biosynthesis process
EDN1	endothelin 1
HPGD	hydroxyprostaglandin dehydrogenase 15-(NAD)
PDPN	podoplanin
PTGIS	prostaglandin I2 (prostacyclin) synthase

Table S8. *Cont.*

Gene Symbol	Gene Description
	Fatty acid metabolic process
EDN1	endothelin 1
HADHB	hydroxyacyl-Coenzyme A dehydrogenase beta subunit
PDPN	podoplanin
PTGIS	prostaglandin I2 (prostacyclin) synthase
PRKAG1	protein kinase, AMP-activated, gamma 1 non-catalytic subunit
	bta-miR-29b
	Fatty acid biosynthetic process
LPL	lipoprotein lipase
PLP	proteolipid protein RG Bos taurus

bta-miR-21*, sequence is from the opposite arm of bta-miR-21.

Table S10. Primer sequences of candidate target genes for qRT-qPCR.

Gene Name	Primer Sequence (5′-3′)	Size (bp)	Annealing Temperature (°C)
ELOVL5	F: GTGGAGGAGAAGCGGACA<br>R: TTGCGGAGGATGAAGAAGA	100	60
ALOX15	F: TGGACTGGCCCTACGAATAC<br>R: GATGGAAGTTGGGAAGAGGA	124	60
ELOVL6	F: CCTCCATCTCAAAGGACTGC<br>R: GGCCTTCTCTCTACCTCTGG	101	60
SC4MOL	F: CTGGGTGACTGTTCGTTTGA<br>R: ATGTGGTGGAAATCGTGATG	120	60
PTGS2	F: GGGCGATGAGCAGTTGTT<br>R: GCAGCAATACGGTTCTGGTA	168	60
PRKAG3	F: CTCCGCTTCTGGATTACCTG<br>R: GTGACAAAGTCGGGAGGAAC	101	60
QKI	F: GAACTCCTCACCCTACTGCTG<br>R: TAGCCACCGCACCTAATACA	150	60
SC5DL	F: TGGAAGATCCCAACTCCATT<br>R: AAGCCCAGGATGTCTTGTGT	118	60
PRKAA1	F: CGCCATACCCTTGATGAATTA<br>R: ATCATTTGGCCGACTTTGAC	102	60
UCP3	F: CACCTGCTCACCGACAACT<br>R: CATATACCGCGTCTTCACCA	175	60
ALOX5AP	F: ACGAAAGCAAGACCCACAAT<br>R: TACATCAGCCCAGCAAAGG	165	60
GST	F: GGACAGATGACAGGGTGGA<br>R: TCGTGTGGTAGATTCGTGCT	142	60
PTGS1	F: GTGCCATCCGAACTCCAT<br>R: AAGGTTGAAGCCCACATCAC	100	60
LPL	F: ACACTTGCCACCTCATTCCT<br>R: TACATTCCTGTCACCGTCCA	124	60
EDN1	F: CTGGACATCATCTGGGTCAA<br>R: GGCATCTCTTCCTGTGGACT	101	60

Table S10. ***Cont.***

Gene Name	Primer Sequence (5′-3′)	Size (bp)	Annealing Temperature (°C)
HADHB	F: TCAACACTCCAGAGCACGTT<br>R: CACACTGGCATCTCTTCCTG	127	60
HPGD	F: AACCTACCTGGGCTTGGATT<br>R: GCGTTCAGTCTCACACCACT	117	60
PDPN	F: TCAGAAAGGTGGCTTGTCAG<br>R: AGGGCGAGTATCTTCCCATT	198	60
PTGIS	F: CAAAGATGGGAAGCGACTG<br>R: GTCAGCACAAGGAACACGAA	122	60
PRKAG1	F: ATCCAGCCTTTGCCTCTGT<br>R: GGACTCAGCCTCCTCCATAC	117	60
CD74	F: ATGAAGAACGCCACCAAGTA<br>R: TTCCAGTCCAAGCCATCC	114	60
SYK	F: CTGGACCCTTTGAGGACTTG<br>R: CTGAGGTTTCTGGCTGATGA	155	60
ADIPOQ	F: AGGGAGAACCTGGAGAAAGTG<br>R: TTGGTAAAGCGAATGGGAAC	110	60
CPT1	F: ACCTGAACGAAGACCTCCAA<br>R: CCACCATGCAAGTTCAGG	100	60
PLP	F: TTCCTGAACGAAGATTCACAA<br>R: AGCTTCGGAAGCAATCGGA	112	60
GAPDH	F: CGTGTCTGTTGTAGGATCTGACCTGC<br>R: CAACCTGGTCCTCTAGTGTAGCCTT	176	58
